# Pathways to family-centered healthcare: co-designing AI solutions with families in pediatric rehabilitation

**DOI:** 10.3389/frobt.2025.1594529

**Published:** 2025-10-30

**Authors:** Silvia Filogna, Giovanni Arras, Tommaso Turchi, Giuseppe Prencipe, Elena Beani, Clara Bombonato, Francesca Fedeli, Gemma D’Alessandro, Antea Scrocco, Giuseppina Sgandurra

**Affiliations:** 1 Department of Developmental Neuroscience, IRCCS Stella Maris Foundation, Pisa, Italy; 2 Department of Computer Science, University of Pisa, Pisa, Italy; 3 Department of Clinical and Experimental Medicine, University of Pisa, Pisa, Italy; 4 FightTheStroke Foundation, Milan, Italy; 5 FTS srl, Milan, Italy; 6 Department of Neuroscience, Psychology, Medication Area and Child Health (NEUROFARBA), University of Florence, Florence, Italy

**Keywords:** artificial intelligence, pediatric rehabilitation, family-centered design, cerebral palsy, participatory design, healthcare technology, ethical AI, stakeholder perspectives

## Abstract

Despite the growing interest in Artificial Intelligence (AI) for pediatric rehabilitation, family engagement in the technologies design remains limited. Understanding how AI-driven tools align with family needs, caregiving routines, and ethical concerns is crucial for their successful adoption. In this study, we actively involved nine families of children with Cerebral Palsy (CP) in an online participatory design workshop, underscoring both the feasibility and the need of integrating family’s perspectives into AI development. Families enthusiastically participated, not only sharing insights but also appreciating the opportunity to contribute to shaping future technologies. Their active engagement challenges the assumption that co-design with families is complex or impractical, highlighting how structured yet flexible methodologies can make such crucial initiatives highly effective. The online format further facilitated participation, allowing families to join the discussion and ensuring a diverse range of perspectives. The workshop’s key findings reveal three core priorities for families: 1. AI should adapt to daily caregiving routines rather than impose rigid structures; 2. digital tools should enhance communication and collaboration between families and clinicians, rather than replace human interaction; and 3. AI-driven systems could empower children’s autonomy while maintaining parental oversight. Additionally, families raised critical concerns about data privacy, transparency, and the need to preserve empathy in AI-mediated care. Our findings reinforce the urgent need to shift toward family-centered AI design, moving beyond purely technological solutions toward ethically responsible, inclusive innovations. This research not only demonstrates the possibility and success of engaging families in co-design processes but also provides a model for future AI development that genuinely reflects the lived experiences of children and caregivers.

## 1 Introduction

The integration of technology in pediatric rehabilitation offers new opportunities to enhance care for children with disabilities. However, the success of such solutions largely depends on their ability to align with family dynamics and daily needs (Kaelin et al., 2021). Despite increasing interest in using technology to support child development, many solutions have traditionally been designed with a clinical focus, often overlooking the central role families play in technology adoption and use in home environments (Herold et al., 2023).

Family-centered care has emerged as a key framework in pediatric rehabilitation, recognizing that family members not only provide primary support to children but also influence therapy adherence and outcomes (Taylor et al., 2004). Other studies highlight that integrating family dynamics into the design of rehabilitation technologies can enhance their effectiveness and foster greater treatment adherence (Dempsey and Keen, 2008; King and Chiarello, 2014). These principles emphasize the need to consider not just clinical efficacy but also how technologies fit within family routines and dynamics. Such approaches align with research suggesting that rehabilitation treatment must go beyond impairment-focused models and promote activity and participation within the family context (Majnemer, 2014).

In recent years, the adoption of participatory design methodologies, which actively engage end-users as co-creators throughout the development process rather than merely as subjects of study, has gained attention as a strategy to involve families in the development of rehabilitation technologies. Approaches such as *co-design* and *design fiction* enable families to contribute their experiential knowledge and contextual insights, ensuring that technological solutions align with real-world needs and seamlessly integrate into daily routines. Previous works have demonstrated that incorporating parents’ perspectives can improve engagement and the effectiveness of digital rehabilitation solutions (Kanitkar et al., 2020), as well as family participation in design, from co-design workshops (Bolster et al., 2021) and focus group (Steinberg et al., 2025) to longitudinal engagement with families (Thiessen et al., 2024), highlighting the importance of understanding family contexts and constraints when designing rehabilitation technologies, particularly for home use. However, clear guidelines on how to systematically integrate the family perspective into technology development are still lacking.

Despite the growing recognition of family-centered approaches in pediatric rehabilitation technology design, the integration of Artificial Intelligence (AI) specifically within participatory design processes involving families remains largely unexplored. The literature shows that while participatory design methods have been successfully applied to develop various digital health tools for pediatric rehabilitation, studies combining family co-design with AI development are extremely scarce. The available research primarily focuses on other technologies such as augmented reality interventions for children with developmental coordination disorder (Welsby et al., 2024), physical activity facilitation tools that explicitly exclude AI components (Bolster et al., 2021), or general digital health platforms without AI. Furthermore, while speculative design methods have been applied in pediatric technology contexts, such as exploring cultural imaginaries of robots with children with disabilities (Stimson, 2024), the combination of design fiction with family-centered AI co-design in rehabilitation settings has not been documented. This gap is particularly significant given that AI systems require different design considerations than traditional digital tools, including algorithmic transparency, adaptive personalisation, and ethical decision-making processes that directly impact family dynamics and caregiving routines.

Addressing this significant literature gap, our study explores how families perceive and envision the role of AI in pediatric rehabilitation, with a particular focus on their involvement in the co-design process of AI-driven solutions. Our work makes several contributions to understanding family perspectives in pediatric rehabilitation: 1. we identify how families need AI systems that can flexibly adapt to their unique daily routines and changing schedules; 2. we reveal how families balance encouraging their children’s independence with maintaining appropriate oversight when using AI-driven tools; 3. we show how families in pediatric rehabilitation face distinctive challenges in integrating new technologies alongside their existing care routines; and 4. we demonstrate a methodological approach for involving families as partners in AI design for pediatric rehabilitation. We adopted the MiniCoDe approach, a participatory design methodology previously used with clinicians to explore AI integration in healthcare (Turchi et al., 2024a), and adapted it specifically for family engagement, aligning it with their unique needs, expectations, and lived experiences with AI technologies. Through a participatory workshop, we gathered insights into families’ experiences and expectations regarding technology use in both home and therapeutic settings. While AI holds potential for personalized rehabilitation (Tsur and Elkana, 2024; Schladen et al., 2020), our primary focus is on co-designing solutions that integrate seamlessly into family routines. By combining participatory design (Turchi et al., 2024b) with design fiction—a method that envisions speculative futures to critically explore possibilities—we examine the intersection of family needs, technological capabilities, and rehabilitative goals.

Our research addresses the following question: *“How can we design AI solutions for pediatric rehabilitation that effectively integrate into family dynamics while supporting rehabilitative goals?”* This study aims at contributing to the development of practical strategies for family-centered design, providing insights for creating technologies that balance rehabilitative effectiveness with usability in real-world family settings.

## 2 Methods

This section begins by outlining our methodological approach, detailing the participatory design process used to engage families in envisioning AI-supported rehabilitation. Afterwards, we present the goals, hypotheses, and description of the study we carried out, following Wohlin et al. (2000)’s guidelines.

### 2.1 Methodology

The MiniCoDe methodology (Malizia et al., 2022), is a workshop-centric approach designed for the ethical deployment of emerging technologies. The MiniCoDe methodology employs two design approaches, Participatory Design and Design Fiction, to gather real input and stimulate thinking about future implications. Specifically, Participatory Design is an approach that emphasizes the active involvement of all stakeholders (especially end-users) in the design process to ensure that the resulting product meets their needs and is usable. Unlike traditional design approaches, where experts create solutions based on assumed requirements, Participatory Design recognizes that users possess unique knowledge about their own experiences and contexts (Sanders and Stappers, 2008). Design Fiction refers to the creation of narrative scenarios that depict how technologies might exist in possible futures, enabling users to reflect on potential implications before implementation (Bleecker, 2022). In our context, Design Fiction allows participants to engage with speculative yet plausible future scenarios of AI-assisted rehabilitation.

We adapted MiniCoDe framework maintaining its core principles and phases while adjusting activities and facilitation approaches for the family context. Our family-specific adaptations included several key modifications: 1. We redesigned the design fiction narrative to center on family routines and emotional dynamics rather than clinical scenarios, featuring a day-in-the-life story that emphasized caregiving challenges and family interactions; 2. We modified brainstorming activities to prioritize families’ experiential knowledge over technical expertise, using prompts that focused on daily caregiving challenges rather than technological specifications; and 3. We incorporated journey mapping of home-based care routines as a core activity, allowing families to visualize AI integration within their natural environments. By presenting a narrative that situates AI within family routines, we encouraged participants to critically reflect on the role of technology, anticipate potential challenges, and co-design meaningful solutions.

The MiniCoDe methodology consists of four key phases:

#### 2.1.1 Prepare phase

Objective: Create a foundation for creative exploration through design fiction that resonates with families’ daily experiences.

This initial phase centers on crafting a narrative that makes abstract AI concepts tangible and relatable for families. We developed a design fiction titled “A Day in 2026: Maria and Luca” (see the Supplementary Material), depicting a plausible future where AI systems support families in managing pediatric rehabilitation. This narrative emphasizes family routines, emotional dynamics, and practical challenges faced in daily care. The design fiction serves as an anchor point, enabling participants to envision how AI might integrate into their existing care practices while considering potential impacts on family life (Bleecker, 2022).

#### 2.1.2 Ideate phase

Objective: Identify key challenges and requirements in daily care routines.

Working in breakout rooms, participants engaged in open brainstorming to identify and document their daily challenges, pain points, and needs in managing pediatric care. Each group then selected their most critical issues for further development. This phase deliberately focused on understanding family needs without constraining thinking to technological solutions, allowing for a deeper exploration of the fundamental challenges families face.

Brainstorming in participatory design differs from conventional ideation by prioritizing diverse voices and lived experiences. We structured this activity to ensure that less technically confident participants could contribute equally, focusing on their expertise in caregiving rather than technological knowledge (Turchi et al., 2024a). This approach acknowledges the experiential authority of families in the rehabilitation process, positioning them as domain experts rather than merely end-users.

#### 2.1.3 Refine phase

Objective: Explore how AI could address identified challenges.

Building on the needs identified in the ideate phase, groups then considered how AI could potentially help address their key challenges. Participants analyzed both benefits and potential concerns of AI solutions, considering factors such as impact on quality of life, data privacy and security, technological reliability, and cost accessibility. This structured evaluation helped bridge the gap between identified needs and potential AI interventions while surfacing important implementation considerations.

During this phase, facilitators provided scaffolding questions such as “How might this solution affect your daily routine?” and “What concerns would you have about using this technology?” to guide systematic evaluation of potential AI solutions. This structured approach helped participants analyze both technical and social dimensions of the proposed technologies, even without specialized technical knowledge.

#### 2.1.4 Reflect phase

Objective: Share and critically examine proposed solutions.

In the final phase, all participants reconvened in the main session where each group presented their developed concept. Groups structured their presentations to address several key aspects: they first described the specific problem their solution aimed to solve, then detailed how their proposed AI solution would function in practice. They also highlighted their solution’s primary anticipated benefits while acknowledging key challenges identified during their evaluation. This structured presentation format enabled systematic comparison of different approaches while facilitating collective learning from each group’s insights and concerns.

This collective reflection phase is critical in participatory design as it allows for the cross-pollination of ideas and surfaces common concerns across different stakeholder perspectives. By facilitating structured feedback across groups, we aimed to identify core requirements and considerations that span diverse family situations.

### 2.2 Research question

Building upon our previous study with clinicians (Turchi et al., 2024b), this research is guided by the overarching question:

“How can we design AI solutions for pediatric rehabilitation that effectively integrate into family dynamics while supporting rehabilitative goals?”

To address this, we explore the following specific questions:

1. How do families envision AI supporting their daily care routines while maintaining family dynamics?
2. What are the key challenges and opportunities in integrating AI into pediatric rehabilitation from a family perspective?

These sub-questions provide a structured approach to investigating the broader inquiry, allowing us to systematically analyze family needs, technological challenges, and the balance between clinical and home-based care.

### 2.3 Study design

We conducted an online workshop implementing the adapted MiniCoDe methodology described in Section 2.1. The design emphasized participatory engagement while accommodating the practical constraints of family participation. By maintaining methodological consistency with our previous clinician study while adapting specific activities for family contexts—such as journey mapping of daily home routines and integrating AI into family care activities—we enabled systematic comparison between stakeholder perspectives.

#### 2.3.1 Settings and tasks

**FIGURE 1:**
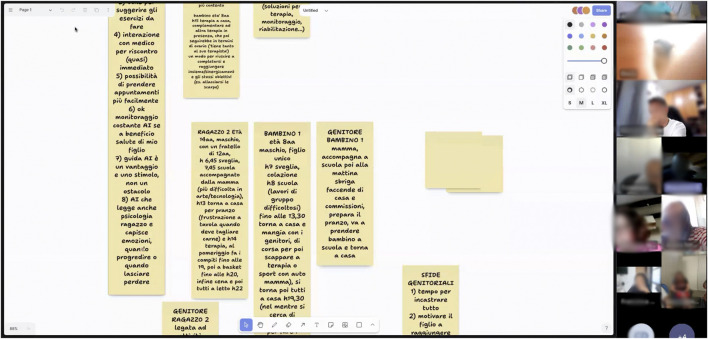
The online workshop setting: participants discussing while using Miro.

The 90-min workshop was conducted online via video conferencing software, utilizing Miro^1^ as the primary collaborative platform, as depicted in Figure 1. The online format was chosen both for practical and methodological reasons: it enabled participation from families across different geographical areas within Italy, reducing travel burden for families already managing complex care schedules, and allowed us to engage participants from diverse socioeconomic backgrounds who might not have been able to attend in-person sessions. This digital setting allowed families to participate from their natural home environment, potentially providing more authentic insights about how AI might integrate into their daily routines, while enabling rich interaction through visual collaboration tools.

#### 2.3.2 Participants

This study, conducted in the framework of the European project AInCP^2^ (Artificial Intelligence in Cerebral Palsy), involved 9 families of children with unilateral CP (of which 1 was also a representative of families association), 4 healthcare professionals, 2 computer scientists/facilitators. Families were recruited from the list of those submitting intent to participate in the AInCP project thanks to the Italian families association (FightTheStroke.org). Recruitment was conducted through direct email contacts with families thanks to their affiliation to FightTheStroke, ensuring voluntary participation and ethical compliance.

Participants were selected based on the age of the children, the geographical area where they live, and the parents’ employment to balance the socioeconomic status of families. The sample was characterized across multiple dimensions to ensure heterogeneity: functional level (assessed using Manual Ability Classification System—MACS and Gross Motor Function Classification System—GMFCS), geographical distribution across Italy (ranging from North to South), and diverse parental occupations. Regarding previous technology experience in rehabilitation, most children (6 out of 9) had no prior exposure to rehabilitation technologies, while 3 had some experience with systems such as VRRS (Virtual Reality Rehabilitation System), robotic upper limb rehabilitation systems, or other experimental platforms. Table 1 provides detailed participant characteristics.

**TABLE 1:** Participant characteristics.

ID	Child age	MACS	GMFCS	Household occupations
1	8 years	2	1	Small business owners
2	14 years	1	1	Law enforcement, unemployed
3	5 years	2	1	Both employed
4	5 years	1	1	Employee, teacher
5	9 years	2	1	Small business owner, employee
6	8 years	2	1	Skilled worker, unemployed
7	7 years	3	1	Employee, unemployed
8	12 years	1	1	Law enforcement, engineer
9	13 years	2	1	Computer scientist, manager

The parents involved represented a range of experiences with technology, from low digital literacy to advanced technical expertise, allowing for a nuanced understanding of family-centered design considerations. This diversity enriched the study by capturing varied expectations, constraints, and aspirations regarding technology use in family settings.

Together with families, four child development specialists, in detail, a child neurologist, two psychologists and a pediatric physical therapist were part of the codesign group and their participation aimed to listen and eventually support, if and when requested, by facilitating the discussion on specific clinical topics and issues. Also, one of the two facilitators was a researcher with lived experience of early-onset childhood disability.

This participant composition allowed us to gather perspectives that reflect diverse family dynamics and caregiving experiences.

Prior to participation, all participants provided informed consent in accordance with established ethical research guidelines.

#### 2.3.3 Procedure

The study followed a structured sequence of sessions, alternating between main plenary discussions and focused breakout activities. This approach ensured that participants could engage in both broad conceptual exploration and detailed design work.

##### 2.3.3.1 Main session: prepare (20 min)

The workshop began with an introductory session where facilitators outlined the objectives and structure of the study.

To establish a shared foundation, we presented a general operational definition of Artificial Intelligence, describing it as “the ability of a computer or machine to imitate human cognitive functions, such as reasoning, learning, and problem-solving”. Examples were provided across healthcare contexts (e.g., image-based diagnostics, remote monitoring, virtual assistants, and epidemic prediction), as well as potential applications in pediatric rehabilitation (e.g., therapy personalization, communication support, mobility assistance). We deliberately avoided technical or model-specific details to prevent biasing participants’ perspectives.

To set the stage for discussion, a speculative design fiction narrative was presented, immersing participants in a plausible future scenario where AI played a central role in family-centered interactions: we created a design fiction titled “A Day in 2026: Maria and Luca” (more details in the Supplementary Material), envisioning a plausible future in which AI systems assist families in managing pediatric rehabilitation.

This narrative served as a provocation, stimulating participants to think beyond existing technologies and consider potential opportunities and challenges. An initial group discussion followed, allowing participants to share their thoughts, expectations, and any preliminary concerns regarding the topic.

##### 2.3.3.2 Breakout rooms: ideate (25 min)

After the main session, participants moved into smaller breakout rooms for a hands-on ideation phase. Using Miro as a collaborative tool, they engaged in journey mapping exercises, visualizing the integration of AI within family interactions. As they constructed these user journeys, they documented key challenges and opportunities, reflecting on both the advantages and limitations of AI systems in this context. The session also marked the beginning of concept development, where participants started outlining potential AI applications that could align with family needs and dynamics.

##### 2.3.3.3 Breakout rooms: refine (25 min)

Building on the initial ideas from the Ideate phase, participants further developed and refined their AI concepts. They expanded on their earlier journey maps, detailing how AI systems could function in specific scenarios. Visual sketching in Miro was used to bring these concepts to life, helping participants articulate their ideas more concretely. Additionally, they considered implementation details, discussing aspects such as adaptability, user control, and how AI might evolve to fit different family contexts over time.

##### 2.3.3.4 Main session: reflect (20 min)

The workshop concluded with a final plenary session where each breakout group presented their developed concepts. This collective discussion allowed participants to compare perspectives, identify recurring themes, and highlight key takeaways. Ethical considerations were also addressed, as participants reflected on potential risks, biases, and the broader impact of AI within family settings. The session provided an opportunity for final reflections, ensuring that the insights generated throughout the workshop were synthesized into meaningful conclusions.

This structured process allowed participants to explore AI integration in a way that was both imaginative and grounded in practical considerations, balancing speculative thinking with concrete design strategies.

### 2.4 Data collection and analysis

**FIGURE 2:**
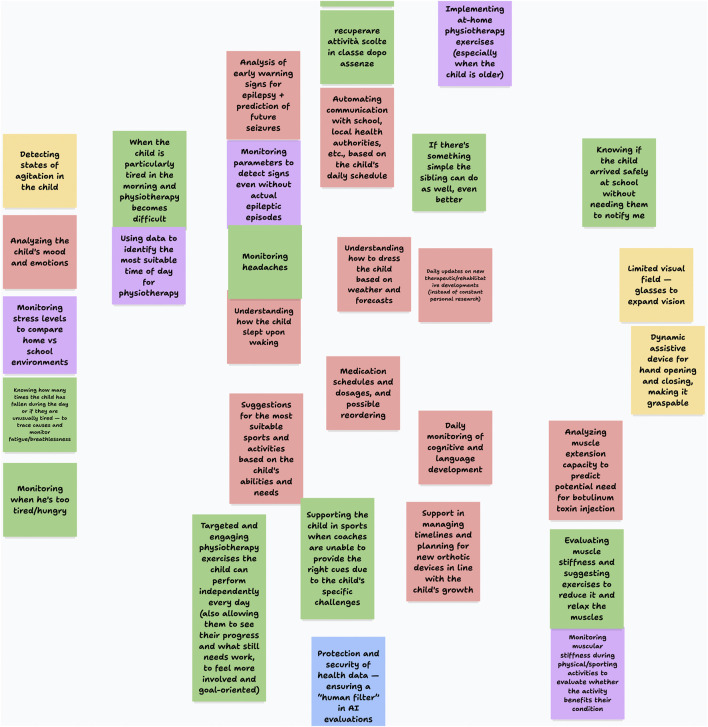
An excerpt from a Miro board created by one of the participants’ group during the workshop (translated).

Throughout the workshop, we collected multiple types of data:

1. Journey maps and design artifacts: All visual materials created by participants in Miro were preserved for analysis (see an excerpt in Figure 2).
2. Discussion notes: Facilitators documented key points raised during both breakout and plenary discussions.
3. Session recordings: With participant consent, all sessions were recorded to facilitate detailed analysis of discussions and interactions.

Data analysis focused on identifying recurring patterns in the journey maps and design concepts created by the participants, with particular attention to common challenges, proposed solutions, and concerns expressed across different family contexts. We catalogued the key features of the AI solutions proposed by each group, along with the primary use cases and implementation considerations they identified.

## 3 Results

The workshop successfully engaged families of children with disabilities, highlighting the feasibility of integrating parents into participatory design processes for AI-driven pediatric rehabilitation. The high level of engagement—reflected in active discussions, critical reflections, and co-created ideas—underscored the value of providing a structured yet flexible environment for families to voice their perspectives. Parents expressed appreciation for the opportunity to contribute meaningfully to technology development, reinforcing the importance of family-centered approaches in designing AI-driven tools.

Following the workshop procedure outlined previously, we conducted a thematic analysis of the digitally recorded sessions and participant feedback. This analysis followed a grounded approach (Pandit, 1996), beginning with the open coding of transcripts to identify a broad range of recurring concepts—such as “family routines”, “privacy concerns”, and “child autonomy”. These codes were iteratively discussed and refined by two researchers, who independently coded a subset of transcripts and then compared their codes to ensure consistency. Any discrepancies were resolved through discussion, fostering a collaborative approach to the coding process. Triangulation was achieved by integrating insights from prior research encounters and field notes, allowing us to corroborate emerging categories across data sources. These concepts were subsequently grouped into *post hoc* thematic categories that capture the most salient issues raised by families. Below, we present four main thematic categories reflecting participants’ perspectives, along with illustrative quotes or examples.

### 3.1 Integrating AI into daily routines

A central theme involved how AI-driven tools could be woven into families’ daily schedules without causing additional strain. Many parents described “busy and rigid timetables”, especially during rehabilitative-intensive periods. While several expressed enthusiasm for automated reminders and digital trackers (e.g., wearable devices or mobile apps that prompt exercise sessions), they also stressed the importance of a flexibile and personlised approach. As one parent noted:

“I do like the idea of a reminder, but it must fit around our unique schedule … sometimes my child needs more breaks than planned” (Parent A).

Another mother emphasized the cascading effects of schedule disruptions:

“They should not move [therapy appointments], because otherwise all the other arrangements fall apart” (Parent B).

The same parent also described the constant rush:

“We’re always running around, really … I would be able to live a less hectic life” (Parent B).

The organizational burden was further illustrated by a third participant:

“I’m full of alarms and reminders on my phone agenda … if you forget, you might get into problems” (Parent C).

Participants suggested that AI-based tools could reduce cognitive load by providing actionable prompts at the right time—*but* only if these systems allow for easy customization and adaptation on the fly.

### 3.2 Enhancing communication with clinicians

Families unanimously recognized the potential benefits of AI in improving communication channels with healthcare professionals. Many described frequent logistical hurdles in scheduling appointments, conveying updates about the child’s condition, and accessing specialized expertise. They advocated for integrated platforms capable of securely sharing progress reports, therapy outcomes, and relevant biometric or emotional data in real time. However, some also voiced concern that increased digitalization might reduce the empathic dimension of clinical care.

“I do not want our doctor to rely solely on data dashboards … We need personal, human contact too” (Parent D), said one caregiver, pointing to the importance of “maintaining a human touch”, especially when sensitive health information is involved.

Another parent highlighted the potential for more immediate medical interaction:

“Interaction with the doctor would be fundamental in my opinion … having the possibility to write two lines to the doctor and them responding almost immediately” (Parent E).

The same parent (Parent D) focused on administrative efficiency:

“Someone who books appointments for me. For example, visits … beyond interaction with the doctor, even just interaction with the switchboard, the secretary” (Parent D).

This highlights the need for AI-driven solutions to complement rather than replace direct communication with clinicians, preserving empathy and trust in healthcare relationships.

### 3.3 Child autonomy and empowerment

A third core category centered on balancing the child’s autonomy with parental oversight. While many parents applauded the idea of AI-driven systems—such as interactive avatars, voice assistants, or gamified exercises—that could motivate children to engage more independently in rehabilitation, they also worried about undue reliance on technology. Several families stated that their children were excited by the possibility of using “fun tech” to track progress or personalize exercises, but parents remained cautious:

“I want my daughter to feel independent, but I’m not sure how much we should trust an AI to suggest new routines or techniques. What if it makes a mistake?” (Parent F).

The emotional challenges of maintaining motivation emerged as another concern: “This year he told me, I will not go to the activity center … they make fun of me … so I start with my stories … to comfort him” (Parent B).

Parents also described the delicate balance required in supporting their children: “When he comes out saying I’ll never be able to do it’ and I have to stay calm … not show my worry, my disturbance at seeing him feel defeated” (Parent E).

Ultimately, families want child-centered innovations that preserve children’s sense of agency while ensuring adult supervision where necessary.

### 3.4 Ethical and privacy concerns

An overarching category that permeated all others was the families’ concern about privacy and ethical issues, particularly regarding data collection and management. Participants frequently cited fears of data breaches, unauthorized data sharing, or a lack of clarity about who would have access to sensitive child-related information.

“We’d love to see how it [the AI] works in the background. If it tells me my child is fatigued, I want to know what data it used to reach that conclusion” (Parent G), remarked one parent, illustrating a desire for more transparency.

However, perspectives on privacy varied considerably. Another participant expressed a more pragmatic view: “I really could not care less … we’re monitored 24/7 by everyone anyway. So I mean, if this serves my son’s health, of course” (Parent E).

A third parent emphasized the importance of human oversight: “Artificial intelligence should always be filtered by a thinking person … I never implement exactly what artificial intelligence tells me” (Parent H).

Concerns about over-surveillance also emerged: “I thought, gosh, but it tells me every day, all these things … about how he slept, how he’s doing … when it becomes pathologizing what is not pathology” (Parent H).

These insights reinforce the necessity of designing AI tools with explicit data protection measures and transparent decision-making processes to build trust among families.

## 4 Discussion

This work represents the second participatory design initiative we have conducted, following the previous workshop where clinicians were the primary stakeholders (Turchi et al., 2024b). By engaging both clinicians and families, we have now gathered insights from two key perspectives that shape the daily experiences of children with CP. We emphasize the importance of beginning the co-design journey by gathering insights from individual stakeholders, even if each provides only a partial view, to assemble the pieces of the broader ecosystem surrounding the child. Specifically, this study highlights the complex interplay between AI-driven healthcare solutions and family-centred design, emphasising the need to align technological capabilities with the lived realities of families in pediatric rehabilitation. Through a participatory design approach, we explored how families envision AI systems supporting their daily routines, fostering child autonomy, and improving communication with clinicians while ensuring ethical and privacy considerations.

Our findings underscore the critical role of family engagement in shaping AI-driven pediatric rehabilitation technologies. By actively involving parents in the design process, we not only captured their lived experiences and expectations but also fostered a sense of ownership over the proposed solutions. This highlights the necessity of shifting from expert-driven technological development toward more inclusive, family-centered approaches. Future work should explore how to scale and refine these participatory methods to ensure long-term impact and usability in real-world clinical and home settings.

### 4.1 Cross-cutting interpretation of findings

The participatory workshop revealed a nuanced understanding of how families perceive and expect AI technologies to integrate into pediatric rehabilitation. Although four distinct themes emerged—integration of daily routine, communication with clinicians, autonomy of children, and ethical concerns—these issues are deeply interconnected and converge on a central message: families highlights the need of AI systems that are supportive, adaptable, and respectful of human relationships. Rather than isolated design features, families envisioned AI as an embedded layer in their care ecosystem, confirming the need for a common point of view among technology experts, clinicians, and families (Bolster et al., 2021; Mackay and Beaudouin-Lafon, 2020). In other words, families want something that can adapt in a flexible way to their lives without replacing human roles (Turchi et al., 2024b).

In addition, the participants consistently highlighted the emotional and relational dimensions of care. Their concerns about empathy, trust, and oversight suggest that technological functionality alone is insufficient. For families, meaningful innovation must be grounded in lived experience, reinforcing the concept of design AI not just for users, but with them. The idea of gamified AI tools, interactive avatars, or voice assistants was welcomed by families as a way to empower their children; however, the findings resonate with broader debates in the literature on Human-Centered AI (HCAI), focusing on adaptive systems that foster autonomy (Bolster et al., 2021) while ensuring appropriate levels of human oversight (Shneiderman, 2020; Buono et al., 2024).

In line with suggestions of Stöger et al. (2021), ethical considerations play a crucial role in the use of AI systems in medicine, from families perspective, as well. In particular, parents express concerns regarding personal data privacy, namely, who can access data, how data will be used, and whether they will have the ability to opt out or modify access rights over time. In addition, families focused on algorithm transparency and trust-building for AI decision-making processes. These concerns highlight the importance of clear communication and participatory governance in the design of future AI systems, highlighting the need for a “privacy by design” approach (Cavoukian et al., 2009).

At the end, the workshop demonstrated that design fiction can be an effective participatory tool. By situating speculative technologies in familiar caregiving narratives, families were able to move beyond surface-level feedback and articulate latent needs, expectations, and ethical boundaries. This underscores the value of narrative-based co-design methods for eliciting input from non-technical users.

### 4.2 Family vs. clinical perspective: a preliminary analysis

The workshops with clinicians (Turchi et al., 2024b) revealed complementary yet distinct perspectives on the role of AI in pediatric rehabilitation. Clinicians focused on how AI could improve diagnostic precision, personalize therapies, and reduce administrative burdens, while stressing the need for ethical safeguards, algorithmic transparency, and human oversight. In contrast, families emphasized the integration of AI into everyday caregiving, prioritizing flexibility, emotional resonance, and trust. Families viewed AI not only as a clinical tool, but also as a potential tool in supporting their children’s routines and autonomy.

Divergences also emerged around autonomy and ethics. Clinicians viewed autonomy through the lens of patient compliance and therapeutic monitoring, whereas families saw it as the child’s empowerment, self-expression and the long-term preservation of their independence over time. Similarly, while clinicians were concerned with institutional responsibilities, such as algorithmic fairness and data governance, families focused on individual control over data and privacy in daily life.

Despite these differences, both groups agreed on key principles: AI systems should be adaptive, explainable, and designed to augment—not replace—human interaction. Clinicians valued empathy in decision-making, while families feared that over-reliance on AI might erode personal relationships with healthcare providers.

Together, these perspectives underscore the need for AI systems that balance clinical effectiveness with usability, emotional sensitivity, and ethical integrity—bridging professional standards with the lived realities of families.

It is important to note that this represents a preliminary comparative analysis based on separate workshops conducted with different stakeholder groups. Future work will involve a more structured and systematic comparative analysis, potentially including joint workshops that bring together clinicians, families, and other stakeholders to explore how these different perspectives can be reconciled and integrated into cohesive AI design frameworks for pediatric rehabilitation.

### 4.3 Design implications for family-centered AI

Considering the raised points, our findings can be translated into concrete recommendations for developers and researchers building AI systems in pediatric rehabilitation. The resulting insights are as follows:

1. Adaptive AI for Dynamic Family Contexts: Families experience varying daily routines, requiring AI tools that can seamlessly adapt to fluctuating schedules, changing needs, and individual caregiving styles. Future systems should learn from behavioral patterns and autonomously adjust therapy schedules and recommendations rather than require manual reconfiguration. *Designers should integrate capabilities that enable real-time adaptation based on family routines and the child’s response patterns.*
2. Transparent and Explainable AI: Trust is a critical factor in the adoption of AI. Families expressed concerns about opaque decision-making processes, particularly regarding child monitoring and therapy recommendations. They wanted to understand the rationale behind AI-generated suggestions and the data sources used in decision-making. *AI systems should provide clear feedback to caregivers, ensuring that users understand how and why AI-generated recommendations are made.*
3. Balancing Child Autonomy and Parental Oversight: AI-driven interventions should be designed to promote children’s engagement and independence without diminishing parental involvement. Families envisioned AI systems that could cleverly assess when children are ready for more independence while maintaining appropriate parental oversight. *Interactive, gamified elements powered by AI can encourage child participation and autonomy by adapting to individual preferences and progress, while configurable parental controls should enable caregivers to set appropriate boundaries.*
4. Human-AI Collaboration in Clinical Communication: Families desire AI-driven communication enhancements that can smartly synthesize progress data and identify patterns worth sharing with clinicians, moving beyond simple data collection to provide contextual insights. However, they emphasized maintaining direct human contact with healthcare providers, requiring a hybrid approach that integrates AI-based efficiency with human empathy. *AI tools should enhance rather than replace human interactions in the healthcare process.*
5. Ethical AI and Data Privacy Measures: Families were particularly concerned about data security and ethical AI use. They wanted transparency about how AI algorithms process their children’s sensitive information and make decisions that affect care routines. Building trust requires clear communication of privacy safeguards and mechanisms for families to control data access and AI decision-making authority. *Developers must prioritize robust data protection, consent-based AI interactions, and transparent data sharing policies.*

By incorporating these principles into AI design, developers can create systems that effectively support both therapeutic objectives and family-centered care, ultimately improving long-term engagement and acceptance.

### 4.4 Theoretical reflections and future research directions

Our findings align with broader trends in HCAI and value-sensitive design, where stakeholder engagement is essential to mitigate risks of algorithmic opacity and socio-technical misalignment. The emphasis on transparency and adaptability reflects calls in the literature to embed accountability and participation into AI systems from the earliest design stages (Floridi and Cowls, 2019; Shneiderman, 2020). In addition, the desire for algorithmic explainability is also particularly strong; parents wanted to understand the rationale behind AI-generated recommendations, rather than passively accepting them as black-box outputs. A co-design approach to developing such solutions is undoubtedly a valuable path to enhancing trust in AI systems (Filogna et al., 2024).

In line with previous co-design research in pediatric rehabilitation (Bolster et al., 2021; Steinberg et al., 2025), the present study confirms that families possess unique contextual knowledge that can enhance the relevance and usability of health technologies. However, our approach extends this work by introducing structured, speculative, and ethically oriented design processes, adapted for remote participation and cross-disciplinary facilitation.

Future research should expand this work by testing and validate AI prototypes in real-world settings, conducting longitudinal studies on AI adoption, and further investigating ethical considerations in AI-driven pediatric rehabilitation. Furthermore, exploring hybrid workshop formats that combine online accessibility with the richness of in-person interaction and developing generalizable methodological toolkits for ethically grounded participatory AI design should be considered.

### 4.5 Threats to validity

While this study provides valuable insights, several factors may influence the validity and generalizability of the findings:

- The study involved a limited number of families, which may not fully capture the diversity of experiences and challenges across different socioeconomic, cultural, and geographic contexts. Future research should expand participant diversity to ensure broader applicability.
- The online workshop format, while effective for remote participation, may have influenced engagement levels. Certain nuances of in-person interactions, such as non-verbal cues and spontaneous discussions, may not have been fully captured. A hybrid study incorporating both online and in-person sessions could provide richer insights.
- Since findings are based on participants’ perceptions and discussions, there is a possibility of response bias. Families may have focused on immediate concerns rather than long-term AI adoption challenges. Complementing qualitative insights with longitudinal studies or real-world AI trials could help validate results.
- The study primarily explores how families imagine AI integration, rather than assessing actual AI system performance. Future studies should incorporate prototype testing to evaluate real-world usability, effectiveness, and unintended consequences.

Despite these limitations, the findings offer a crucial starting point for designing AI solutions that align with both family and clinical needs. Addressing these threats to validity in future work will strengthen the reliability of AI-driven healthcare recommendations.

## 5 Conclusion

This study emphasizes that engaging families in the participatory design of AI-driven pediatric rehabilitation tools is important, feasible, and highly valuable. The workshop showcased active and enthusiastic participation, with parents not only contributing insights but also expressing appreciation for being included in shaping future technologies. The success of this initiative, driven by the families’ active collaboration with researchers, highlights how structured yet flexible methodologies can effectively facilitate engagement, even in people who may face logistical barriers to participation. Indeed, the online format played a key role in enhancing accessibility, allowing families to share their experiences and perspectives from their own homes. In addition, the presence of a researcher with lived experience was highly appreciated, as he was regarded as a representative of their future child. These findings suggest that designing AI-based interventions with families is not only possible but essential to ensure that technological solutions align with real-world caregiving dynamics.

To truly design a technology that is co-created with all stakeholders, a comprehensive, multi-stakeholder approach would allow us to identify common priorities, potential conflicts, and areas for synergy, ultimately ensuring that AI-driven solutions are not just tailored to one group’s needs but holistically embedded into the child’s life and care network.

Future research on this topic will contribute to the broader effort of democratizing AI-driven healthcare, advocating for solutions that are not only technologically robust but also deeply tuned to the human experience of care. By ensuring that AI systems are inclusive, adaptable, and transparent, we can bridge the gap between innovation and real-world impact, fostering healthcare solutions that truly serve all stakeholders involved in pediatric rehabilitation.

## Data Availability

The original contributions presented in the study are included in the article/Supplementary Material, further inquiries can be directed to the corresponding authors.

## Footnotes

1
https://miro.com

2
https://www.aincp.eu/

## References

[B1] Bleecker J. (2022). Design fiction: a short essay on design, science, fact, and fiction. Mach. Learn. city Appl. Archit. urban Des., 561–578. 10.1002/9781119815075.ch47

[B2] Bolster E. A. M. Gessel C. V. Welten M. Hermsen S. Lugt R. V. D. Kotte E. (2021). Using a co-design approach to create tools to facilitate physical activity in children with physical disabilities. Front. Rehabilitation Sci. 2, 707612. 10.3389/fresc.2021.707612 PMC9397745 36188842

[B3] Buono P. Berthouze N. Costabile M. Grando A. Holzinger A. (2024). Special issue on human-centered artificial intelligence for one health. Artif. Intell. Med. (2017). 156, 102946. 10.1016/j.artmed.2024.102946 39198042

[B4] Cavoukian A. (2009). Privacy by design: the 7 foundational principles. Inf. Priv. Comm. Ont. Can. 5, 12.

[B5] Dempsey I. Keen D. (2008). A review of processes and outcomes in family-centered services for children with a disability. Top. Early Child. Special Educ. 28, 42–52. 10.1177/0271121408316699

[B6] Filogna S. Malizia A. Mazzei D. Prencipe G. Sgandurra G. Turchi T. (2024). “Telemedicine and ai: from co-design to explainability,” in 2024 IEEE 8th forum on research and technologies for society and industry innovation (RTSI), 363–368. 10.1109/RTSI61910.2024.10761199

[B7] Floridi L. Cowls J. (2019). A unified framework of five principles for AI in society. Harv. Data Sci. Rev. 1. 10.1162/99608f92.8cd550d1

[B8] Herold L. Bosques G. Sulzer J. (2023). Clinical uptake of pediatric exoskeletons. Am. J. Phys. Med. and Rehabilitation 103, 302–309. 10.1097/PHM.0000000000002371 38063305

[B9] Kaelin V. Valizadeh M. Salgado Z. Parde N. Khetani M. (2021). Artificial intelligence in participation-focused pediatric rehabilitation: a scoping review. Archives Phys. Med. Rehabilitation 102, e111. 10.1016/j.apmr.2021.07.451 PMC8603165 34734833

[B10] Kanitkar A. Parmar S. Szturm T. Restall G. Rempel G. Sepehri N. (2020). Parents’ perspectives on a computer game–assisted rehabilitation program for manual dexterity in children with cerebral palsy: qualitative analysis of expectations, child engagement, and benefits. JMIR Rehabilitation Assistive Technol. 8, e24337. 10.2196/24337 34057424 PMC8204242

[B11] King G. Chiarello L. (2014). Family-centered care for children with cerebral palsy. J. Child Neurology 29, 1046–1054. 10.1177/0883073814533009 24810084

[B12] Mackay W. E. Beaudouin-Lafon M. (2020). Participatory design and prototyping. Springer International Publishing, 1–33. 10.1007/978-3-319-27648-9_31-1

[B13] Majnemer A. (2014). Innovation toward better living. J. Child Neurology 29, 1028–1029. 10.1177/0883073814535505 24870367

[B14] Malizia A. Carta S. Turchi T. Crivellaro C. (2022). “Minicode workshops: minimise algorithmic bias in collaborative decision making with design fiction,” in Proceedings of the hybrid human artificial intelligence conference.

[B15] Pandit N. R. (1996). The creation of theory: a recent application of the grounded theory method. Qual. Rep. 2, 1–15. 10.46743/2160-3715/1996.2054

[B16] Sanders E. B.-N. Stappers P. J. (2008). Co-creation and the new landscapes of design. Co-design 4, 5–18. 10.1080/15710880701875068

[B17] Schladen M. Cleary K. Koumpouros Y. Monfaredi R. Salvador T. Talari H. F. (2020). Toward evaluation of the subjective experience of a general class of user-controlled, robot-mediated rehabilitation technologies for children with neuromotor disability. Inf. (MDPI) 7, 45–50. 10.3390/informatics7040045 34522643 PMC8436173

[B18] Shneiderman B. (2020). Human-centered artificial intelligence: three fresh ideas. Trans. Human-Computer Interact., 109–124. 10.17705/1thci.00131

[B19] Steinberg P. Gefen N. Weiss P. L. Beeri M. Landa J. Krasovsky T. (2025). What does “tele” do to rehabilitation? thematic analysis of therapists’ and families’ experiences of pediatric telerehabilitation. Disabil. Rehabilitation, 1–9. 10.1080/09638288.2025.2496355 40294907

[B20] Stimson C. E. (2024). Exploring cultural imaginaries of robots with children with brittle bone disease: a participatory design study. Med. Humanit. 50, 705–714. 10.1136/medhum-2024-013039 39746718 PMC11877108

[B21] Stöger K. Schneeberger D. Holzinger A. (2021). Medical artificial intelligence: the European legal perspective. Commun. ACM 64, 34–36. 10.1145/3458652

[B22] Taylor N. Dodd K. Mcburney H. Graham H. (2004). Factors influencing adherence to a home-based strength-training programme for young people with cerebral palsy. Physiotherapy 90, 57–63. 10.1016/J.PHYSIO.2003.09.001

[B23] Thiessen R. Geiskkovitch D. Y. Dabiri M. Berzuk J. M. Lo N. Sakamoto D. (2024). “Understanding family needs: informing social robot design to support children with disabilities to engage in play,” in Proceedings of the 12th international conference on human-agent interaction, 71–80. 10.1145/3687272.3688301

[B24] Tsur E. E. Elkana O. (2024). Intelligent robotics in pediatric cooperative neurorehabilitation: a review. Robotics 13, 49. 10.3390/robotics13030049

[B25] Turchi T. Malizia A. Borsci S. (2024a). Reflecting on algorithmic bias with design fiction: the MiniCoDe workshops. IEEE Intell. Syst. 39, 40–50. 10.1109/MIS.2024.3352977

[B26] Turchi T. Prencipe G. Malizia A. Filogna S. Latrofa F. Sgandurra G. (2024b). Pathways to democratized healthcare: envisioning human-centered ai-as-a-service for customized diagnosis and rehabilitation. Artif. Intell. Med. 151, 102850. 10.1016/j.artmed.2024.102850 38555849

[B27] Welsby E. Hobbs D. Hordacre B. Ward E. Hillier S. (2024). Co-design for technology in paediatric therapy: developing an augmented reality intervention for children with developmental coordination disorder. J. Rehabilitation Assistive Technol. Eng. 11, 20556683241266780. 10.1177/20556683241266780 39132468 PMC11311161

[B28] Wohlin C. Runeson P. Höst M. Ohlsson M. C. Regnell B. Wesslén A. (2000). Experimentation in software engineering: an introduction. Norwell, MA, USA: Kluwer Academic Publishers.

