# Supplementary material for: Pathways to family-centered healthcare: co-designing AI solutions with families in pediatric rehabilitation
Source: Front Robot AI. 2025 Oct 30;12:1594529. doi: 10.3389/frobt.2025.1594529 (PMC12611681; doi:10.3389/frobt.2025.1594529)
Supplement: Supplementary file 1 [file Supplementaryfile1.pdf]

Supplementary Material

1 DESIGN FICTION NARRATIVE

NARRATOR: It's dawn in 2026. We meet Maria, mother of Luca, a lively 8-year-old boy with cerebral palsy.

MARIA: Good morning, Aida. How is Luca this morning?

AIDA (AI): Good morning, Maria. The sensors indicate Luca slept well. His posture is optimal, and his vital signs are normal.

MARIA: Excellent! Which physical therapy exercises are recommended for today?

AIDA (AI): Based on Luca's progress, the doctor suggests 15 minutes of assisted stretching, followed by 20 minutes of balance exercises using our augmented reality system.

LUCA: Mum, look! I got all the stars.

MARIA: Great job, Luca! Aida, can you show me Luca's progress from the last week?

AIDA (AI): Certainly! Luca has improved his balance by 12% and his muscle strength by 8%. I suggest informing Dr. Bianchi about these improvements.

AIDA (AI): Maria, it's time for Luca's medication. I've already adjusted the dosage based on his latest tests, as approved by the doctor.

MARIA: Aida, Luca seems a bit frustrated with his speech exercises.

AIDA (AI): I understand. Based on previous data, Luca responds better when the exercises are integrated into his favorite games. Shall I adapt the communication app to include characters from his favorite cartoon?

MARIA: That would be perfect! Thank you!

NARRATOR: As the day draws to a close, Maria reflects on how technology has transformed their daily life.

MARIA: Aida, can you summarize today's activities?

AIDA (AI): Certainly! Maria, today Luca made significant progress in his exercises, maintained a good medication routine, and his mood was generally positive. I've scheduled a video call with Dr. Bianchi tomorrow to discuss these recent improvements.

NARRATOR: This is a day in the life of Maria and Luca, where technology doesn't replace human affection but enhances its effectiveness.
